# The progress and trends of the mechanism of cardiopulmonary bypass-associated acute lung injury: A narrative review

**DOI:** 10.1097/MD.0000000000043019

**Published:** 2025-06-27

**Authors:** Hang Gao, Tingting Yi, Bo Hu, Zhiquan Lv, Linyu Ma, Shouyong Wang

**Affiliations:** a Department of Anesthesiology of the People’s Hospital of Yongchuan District, Chongqing, China; b Department of Anesthesiology of the Affiliated Yongchuan Hospital of Chongqing Medical University, Chongqing, China; c Nursing Department, The Affiliated Yongchuan Hospital of Chongqing Medical University, Chongqing, China.

**Keywords:** acute lung injury, cardiopulmonary bypass, glycocalyx, macrophages, neutrophils, therapeutics

## Abstract

Studies into the mechanisms of cardiopulmonary bypass-associated acute lung injury have not presented breakthroughs for many years, resulting in the stagnation of management strategies and clinical medicine measures. This is a key factor affecting the prognoses of open-heart surgery patients. Future studies should focus on key targets of inflammation, such as neutrophils, macrophages, and the glycocalyx cell coat, and further explore advanced technologies, such as gene editing and single-cell sequencing, to reveal the underlying mechanisms of cardiopulmonary bypass-associated acute lung injury and to find effective prevention and treatment strategies.

## 1. Introduction

Open-heart surgery involving cardiopulmonary bypass (CPB) remains the gold standard and most effective approach for correcting various congenital cardiovascular malformations and for rescuing patients with organic heart disease. Nevertheless, acute lung injury (ALI) frequently emerges as an inevitable complication post-surgery. In mild cases, this may present as a systemic inflammatory response syndrome or compromised lung ventilation function, while severe cases can progress to acute respiratory distress syndrome (ARDS). Although the incidence of multiple organ dysfunction syndrome and multiple organ failure is <3%,^[1–3]^ the mortality rate associated with multiple organ failure can be as high as 30%. Despite advancements in understanding the mechanisms of CPB-induced lung injury over the past decades, the efficacy of traditional preventive and therapeutic strategies has plateaued. This narrative review consolidates recent literature, particularly at the cellular and molecular levels, to outline the current progress in this field and to suggest future research directions.

## 2. Materials and methods

We performed an online literature search on PubMed, Google Scholar, CNKI (China National Knowledge Infrastructure) and Web of Science from inception through December 2023, focusing on studies related to ALI associated with CPB. Our search incorporated the following Medical Subject Headings terms: “CPB” OR “extracorporeal circulation” AND “ALI” AND “lung injury.” This search generated approximately 900 results. We systematically excluded duplicated studies, those with unrelated topics, those featuring inappropriate study designs, those with inaccessible full texts, and those providing incomplete data. Ultimately, 29 articles were selected and cited in this narrative review.

## 3. Discussions/observations

### 3.1. Research status of extracorporeal circulation-associated lung injury

The main pathological characteristics of CPB-associated ALI are the infiltration of various inflammatory cells into the lung and the dysfunction of alveolar ventilation. Previous studies suggest that the key mechanisms of CPB-associated ALI include blood monocyte activation due to contact between blood and the nonphysiologically interface and pulmonary ischemia-reperfusion injury. Other stressors, including heparin anticoagulation and neutralization, transfusion of blood and biological products, blood cooling and rewarming, changes in the shear stress levels of blood cells, endotoxin translocation, etc, are also involved to some degree.^[4]^ In the past few decades, there have been no breakthroughs in research into the mechanisms of CPB-associated ALI, resulting in relatively limited clinical strategies and measures, which have been key factors affecting the prognosis of open-heart surgery with CPB for many years. Among existing intervention strategies, improvements in biomaterial compatibility and improvements in surgery and perfusion techniques have achieved certain effects, while protective lung ventilation strategies, leukocyte filtration, ultrafiltration, protease and cytokine inhibition, hormones, intestinal flora regulation and other measures have shown limited or even controversial effects.^[5]^ This indicates that the current understanding of the mechanism of CPB-associated ALI needs to be expanded to find new targets for intervention and more specifically targeted intervention measures.

### 3.2. Possible role of neutrophils in lung injury associated with CPB and its management

#### 3.2.1. Involvement of neutrophils in CPB-associated lung injury

Neutrophil granulocytes are the most abundant nucleated cells in circulating blood and are the main component of the body’s nonspecific cellular immune function. Pulmonary infiltration of neutrophils is regarded as the signature event of lung injury due to various causes, regardless of whether the cause is infectious or noninfectious.^[6]^ Studies have found that CPB can lead to a large amount of neutrophil infiltration in lung tissues^[7]^ and significantly increase the gene expression levels, protein concentrations and hydrolytic activities of elastase and matrix metalloproteinase in blood, lung tissue, and bronchoalveolar lavage fluid, accompanied by corresponding tissue and function damage such as edema and hypoxemia.^[8]^ The administration of neutrophil elastase inhibitors can reduce systemic inflammatory response syndrome and lung function impairment caused by CPB.^[9]^ Other researchers have found that CPB can cause mobilization of a large number of neutrophils from bone marrow to peripheral blood circulation, doubling the number and oxidation capacity of lung neutrophils, accompanied by a decrease in lung oxygenation function. These phenomena can be significantly improved with the administration of β2 integrin CD18 monoclonal antibody.^[8]^ These studies have shown that CPB results in an overall enhancement of neutrophils in aspects of bone marrow mobilization, activation, function, and release of active substances.

#### 3.2.2. Neutrophils as targets for the prevention and treatment of CPB-associated lung injury

Generally, no pathogens or other foreign substances invade the lungs during CPB; it could be that the enzymes and other bioactive components carried by neutrophils invading the lung are the key factors for ALI. Animal experiments have shown that filtering peripheral blood leukocytes has a certain preventive effect on lung injury caused by CPB. Bando et al found that filtering peripheral white blood cells can reduce the peripheral white blood cell count by 97%, significantly inhibit the generation of oxygen free radicals and the activation of complement, reduce the content of extravascular pulmonary fluid, improve pulmonary oxygenation function, and thus increase the patient survival rate.^[10]^ However, other clinical and experimental studies have shown that the effectiveness of this physical filtration method in preventing lung injury caused by CPB is limited,^[11]^ mainly because it cannot prevent lung ischemia – reperfusion damage to the lung caused by CPB or the release of reserve white blood cells from the bone marrow and lymphatic system, nor can it reverse the function of activated neutrophils infiltrating the vasculature. This suggests that further understanding of the key mechanism of neutrophil pulmonary infiltration during CPB is needed; and on this basis, targeted blocking of neutrophil pulmonary infiltration and activation caused by CPB may be a more effective means to prevent and treat CPB-associated ALI.

#### 3.2.3. Modulation of the SDF-1/CXCR4 signaling axis to regulate neutrophil infiltration for the prevention and treatment of CPB-associated ALI

Stromal cell-derived factor-1 (SDF-1), a CXC subfamily chemotactic protein derived from bone marrow stromal cells, is widely expressed in various tissues throughout the body. It has chemotactic effects on lymphocytes, monocytes, neutrophil granulocytes, dendritic cells and CD34+ progenitor cells and is very important for tissue growth, development and differentiation and the inflammatory response. C-X-C chemokine receptor 4 (CXCR4) is a G-protein coupled receptor consisting of 352 amino acids and comprising 7 transmembrane segments. CXCR4 is widely expressed on the surface of blood, immune and central nervous system cells. SDF-1 and CXCR4 are both members of the CXC subfamily and are natural ligands and receptors of one another, forming the SDF-1/CXCR4 signaling axis, which plays an important role in various biological processes related to cell migration.^[12]^ In CPB-associated ALI, inflammatory cell infiltration into the lung mainly comprising neutrophils is a basic pathophysiological change. Whether the SDF-1/CXCR4 signaling pathway is involved in this mechanism is a question worth studying. Our study found that CPB could significantly increase plasma SDF-1 levels and CXCR4 expression in neutrophils in children undergoing ventricular septa repair with CPB, the chemotactic effect between plasma and neutrophils was significantly enhanced after CPB, and this enhancement could be blocked by the CXCR4-specific antagonist AMD3100.^[13]^ After CPB, the expression and phosphorylation level of plasma CXCR4 can be significantly upregulated in neutrophils cultured in vitro and thereby enhance its chemotactic ability,^[14]^ suggesting that the SDF-1/CXCR4 mechanism is involved in neutrophil lung infiltration during CPB. In addition, in a separate study, we used CRISPR/Cas9 technology to construct an inducible expression gene editing vector targeting CXCR4 and demonstrated its gene editing efficiency and ability to regulate cell chemotaxis in the human gastric cancer cell line MKN-45,^[15]^ which suggests that it is feasible to regulate the movement of neutrophils by modulating SDF-1/CXCR4 signals using a gene editing technique. However, whether regulating SDF-1/CXCR4 signals to reduce neutrophil lung infiltration can alleviate CPB-associated ALI needs to be further investigated.

### 3.3. Targeting macrophages to prevent lung injury during CPB

#### 3.3.1. Possible role of macrophage metabolic reprogramming in CPB-associated ALI

Macrophages are an important part of and the first line of defense in the body’s innate immune system. Macrophage polarization (metabolic reprogramming) plays a key role in the occurrence, development and outcome of ALI for various reasons.^[16,17]^ Macrophages can independently recognize and phagocytose cells that are senescent, mutated or infected by foreign microorganisms; present internal and external antigens to lymphocytes through MHC class I and class II molecules; sensitize and activate lymphocytes; synthesize and secrete a variety of cytokines to stimulate and maintain immune activity; and recruit neutrophils and other inflammatory cells through intercellular communication. Macrophages thus play a central role in the body’s immune system.^[16–18]^

#### 3.3.2. Demonstration of macrophage heterogeneity at the single-cell level further reveals the pathogenesis of CPB-associated ALI

Single-cell heterogeneity is determined by lineage development, and the past cellular state determines the current and future developmental potential, which contains valuable information on physiological and pathological mechanisms at the molecular and cellular levels. At certain space-time nodes, each cell has a unique contribution to the overall physiological or pathological state of a tissue or organ. In previous cell population studies, macrophages were classified by dichotomy, and the gene and protein variants were investigated by batch average methods, which do not accurately group or define the heterogeneity of macrophages under inflammatory conditions and miss valuable information at the cellular and molecular levels. Omics technology can make up for these shortcomings, and it can reveal the dynamic changes and rules of macrophage heterogeneity at the single-cell level; thus, it is of great value to elucidate the underlying mechanisms of the occurrence, development and outcome of the inflammatory response and to explore targeted prevention and treatment strategies for the inflammatory response.

**Figure 1.**
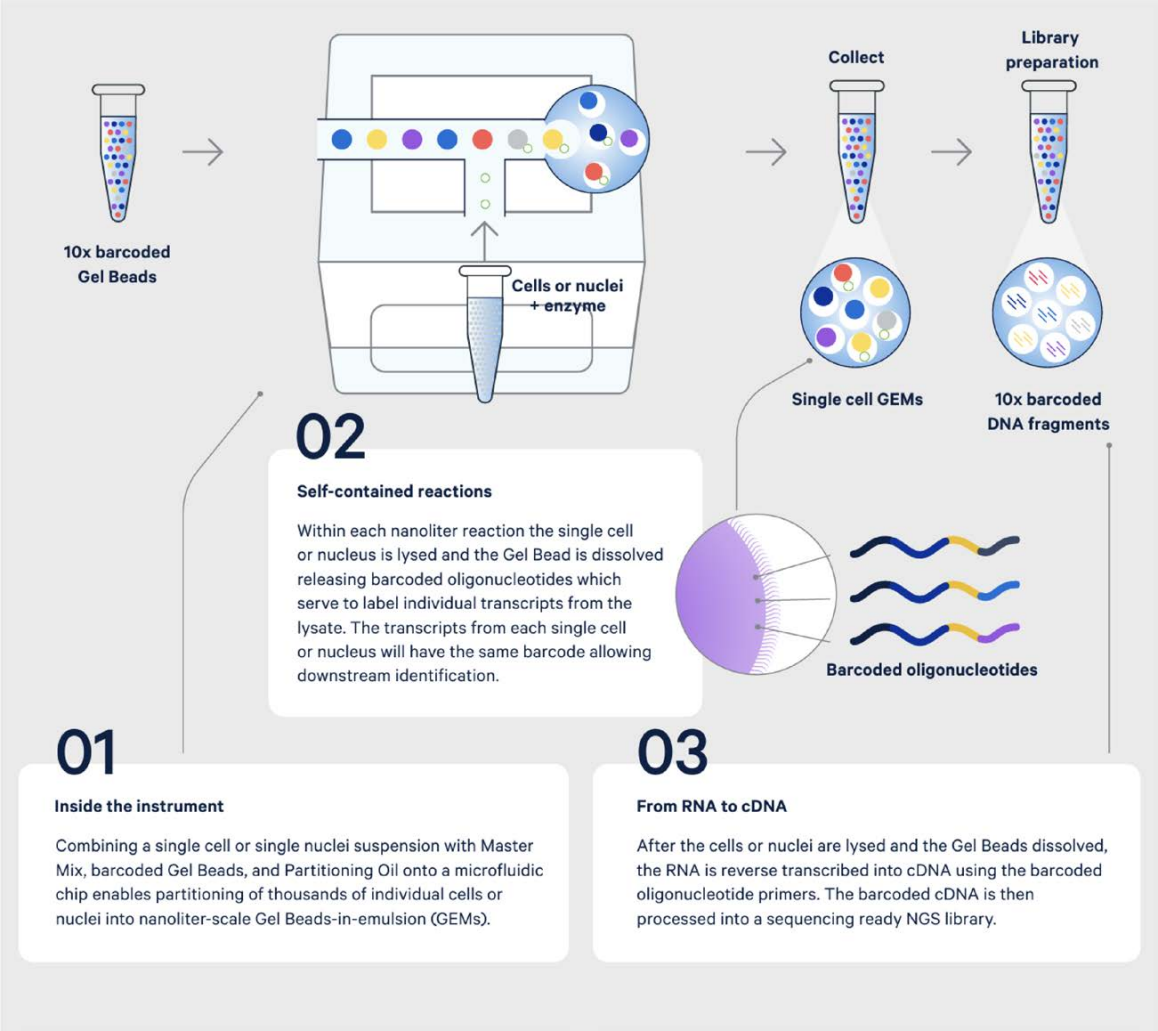
Basic principles of single-cell RNA sequencing (adapted from 10X Genomics official website: https://www.10xgenomics.com/cn). cDNA = complementary DNA, DNA = deoxyribonucleic acid, GEMS = gel bead-in-emulsion, RNA = ribonucleic acid.

Single-cell RNA sequencing (scRNA-seq), the most advanced and commonly used technique in the field of single-cell omics, uses microbeads or microwells to create an independent and tractable RT–PCR system for each cell and uses multiple barcodes to achieve accurate labeling at the single-gene level (Fig. 1). This enables high-throughput single-gene sequencing analysis of tens of thousands of individual cell transcriptomes in a single experiment, enabling the discovery of subtle changes in gene expression at the single-cell level.^[19]^ In the past decade, scRNA-seq has achieved fruitful results in many fields, such as developmental biology, oncology, and epigenetics, and has profoundly explained some key scientific issues in many disciplines. In the field of inflammation, scRNA-seq has been used to draw a map of epithelial cell subsets in an acute chemical lung injury model, and new Sox9-positive type II alveolar epithelial cell subsets with stem cell differentiation potential have been identified that are recruited to the injured epithelial barrier and participate in epithelial cell regeneration and tissue repair.^[20]^ Neutrophils play a key role in the occurrence and development of ARDS. A recent study mapped neutrophil heterogeneity in mouse ARDS models and identified 2 novel neutrophil subgroups with significant heterogeneity in transcription, distribution and function: high expression of Fth1 type (Fth1hi Neu) and Prok2 type (Prok2hi Neu). Clinical sample analysis shows that ARDS patients with high expression of Fth1 and low expression of Prok2 in airway neutrophils may have worse outcomes.^[21]^ These studies fully demonstrate that scRNA-seq can fully disclose the heterogeneity of target cells and advance the understanding of disease pathogenesis at a deeper level. Studies have shown that CPB can cause downregulation of circulating monocyte integrin and adherin receptor expression, upregulation in the proportion of immature monocytes, and foster a close relationship between pulmonary macrophage-derived microvesicles and CPB-associated ALI.^[21–24]^ However, it is still unclear whether there is a close relationship between the single-cell heterogeneity of lung macrophages and CPB-associated ALI, and relevant studies are warranted.

### 3.4. Prevention and treatment of CPB-associated ALI with glycocalyx as a target

**Figure 2.**
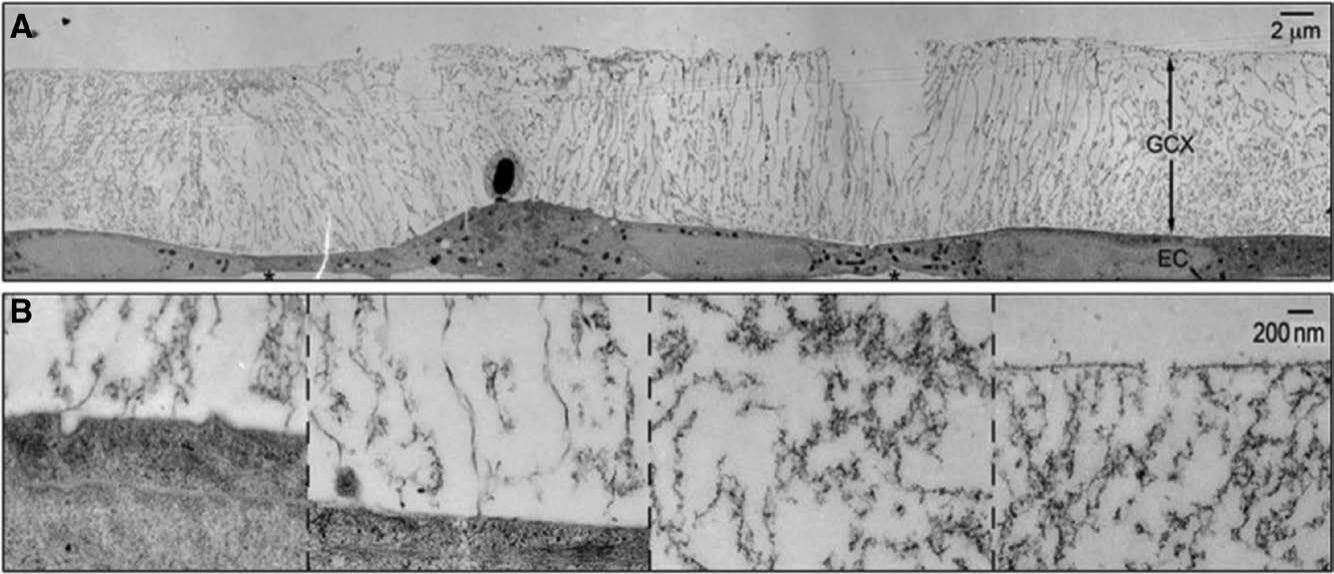
The structure of glycocalyx. (A) Transmission Electron Microscope of glycocalyx preserved by ruthenium red and osmium tetroxide. (B) High-magnification image of glycocalyx.^[25]^ EC = endothelial cell, GCX = glycoclyx.

The glycocalyx is a negatively charged hydration structure composed of polysaccharides and protein macromolecules on the endothelial surface of capillaries, mainly comprising core proteins and side chain structures. Viewed using electron microscopy, the glycocalyx is a hair-like barrier material layer with a thickness of 1 to 2 μm that plays roles in the mechanical barrier, physical filtration, transmembrane signal transduction, coagulation regulation and combatting inflammation (Fig. 2).^[25]^

The integrity of the glycocalyx plays a crucial role in maintaining the selective permeability of capillaries. When the glycocalyx is damaged, vascular permeability and levels of markers, such as those of sydecan-1, heparin sulfate, and hyaluronic acid, are increased in the blood. Studies have demonstrated that heparinase can cause degradation of the glycocalyx and significantly increase pulmonary capillary permeability, while glucocorticoids can reduce pulmonary interstitial edema by maintaining normal permeability through inhibition of the degradation of the glycocalyx.^[12]^ Many studies have confirmed that lung injury is closely related to shedding of the glycocalyx. Under normal physiological conditions, the intact polysaccharide coating has roles in the mechanical barrier, physical filtration, adhesion molecule hiding and other functions. It is impossible for circulating neutrophils to cross the intact polysaccharide coating to contact vascular endothelial cells or undergo transvascular migration.^[26]^ In vivo animal experiments have found that the transvascular migration of neutrophils occurs after the shedding of the polysaccharide coating in an LPS-induced ALI model, and heparinase inhibitors can reduce LPS-induced lung injury by inhibiting the shedding of the polysaccharide coating.^[12]^ CPB was also found to be a potent factor causing the destruction of the glycocalyx. After CPB, the plasma concentrations of sydecan-1, heparin sulfate, and hyaluronic acid increased, and the patient’s sublingual artery perfusion significantly decreased, at the same time.^[27]^ The plasma concentration of syndecan-1 caused by CPB was significantly increased, which was positively correlated with the plasma concentration of major inflammatory cytokines IL-6 and IL-8 but negatively correlated with the plasma concentration of the major anti-inflammatory cytokine IL-10.^[28]^ In addition, syndecan-1 can also led to massive mobilization of neutrophils in bone marrow and cause a significant increase in the number of neutrophils in peripheral blood.^[29]^

### 3.5. Limitation

The objective of this article is to provide a concise overview of the current state of research on CPB-associated ALI, rather than to revise clinical protocols for diagnosis and treatment. Consequently, the focus here is on elucidating the findings from the existing literature, without delving into the classification of the evidence hierarchy and the genres of the published works – a restraint in our current analysis. Nevertheless, we are confident that the insights gathered offer intriguing perspectives and provoke thought in the domain of ALI associated with CPB.

## 4. Conclusions

In summary, there have been no breakthroughs in the research into the mechanisms and coping strategies of CPB-associated ALI for many years. Studies into the key points of CPB-associated ALI occurrence, such as neutrophil infiltration, macrophage reprogramming, and polysaccharide degradation, along with new technologies such as gene editing and single-cell omics may further reveal the underlying mechanisms of CPB-associated ALI and identify effective prevention and treatment strategies.

## Author contributions

**Data curation:** Hang Gao, Tingting Yi, Bo Hu.

**Conceptualization:** Zhiquan Lv, Linyu Ma, Shouyong Wang.

**Formal analysis:** Hang Gao, Tingting Yi, Bo Hu.

**Investigation:** Hang Gao, Tingting Yi, Bo Hu, Zhiquan Lv.

**Methodology:** Shouyong Wang.

**Project administration:** Shouyong Wang.

**Supervision:** Zhiquan Lv, Shouyong Wang.

**Writing – original draft:** Zhiquan Lv, Shouyong Wang.

**Writing – review & editing:** Shouyong Wang.
